# Preclinical assessment on neuronal regeneration in the injury-related microenvironment of graphene-based scaffolds

**DOI:** 10.1038/s41536-021-00142-2

**Published:** 2021-06-02

**Authors:** Yun Qian, Xu Wang, Jialin Song, Wei Chen, Shuai Chen, Yi Jin, Yuanming Ouyang, Wei-En Yuan, Cunyi Fan

**Affiliations:** 1 Department of Orthopedics, Shanghai Jiao Tong University Affiliated Sixth People’s Hospital, Shanghai, China; 2 Shanghai Engineering Research Center for Orthopaedic Material Innovation and Tissue Regeneration, Shanghai, China; 3 Youth Science and Technology Innovation Studio, Shanghai Jiao Tong University School of Medicine, Shanghai, China; 4 Department of Anesthesiology and Perioperative Medicine, Tufts Medical Center, Boston, MA USA; 5 Engineering Research Center of Cell & Therapeutic Antibody, Ministry of Education, and School of Pharmacy, Shanghai Jiao Tong University, Shanghai, China

**Keywords:** Regeneration and repair in the nervous system, Peripheral nervous system

## Abstract

As the application of graphene nanomaterials gets increasingly attractive in the field of tissue engineering and regenerative medicine, the long-term evaluation is necessary and urgent as to their biocompatibility and regenerative capacity in different tissue injuries, such as nerve, bone, and heart. However, it still remains controversial about the potential biological effects of graphene on neuronal activity, especially after severe nerve injuries. In this study, we establish a lengthy peripheral nerve defect rat model and investigate the potential toxicity of layered graphene-loaded polycaprolactone scaffold after implantation during 18 months in vivo. In addition, we further identify possible biologically regenerative effects of this scaffold on myelination, axonal outgrowth, and locomotor function recovery. It is confirmed that graphene-based nanomaterials exert negligible toxicity and repair large nerve defects by dual regulation of Schwann cells and astroglia in the central and peripheral nervous systems. The findings enlighten the future of graphene nanomaterial as a key type of biomaterials for clinical translation in neuronal regeneration.

## Introduction

The graphene-based materials (GBM) are a class of two-dimensional carbon nanomaterials with incredible physical, chemical, and mechanical properties^1–3^. They include graphene and its derivatives, such as graphene oxide (GO), reduced GO, graphite oxide, and ultrathin graphite, and are highly stiff, elastic, thermally, and electrically conductive^4–6^. These qualities make them increasingly important and popular in different areas of life, such as bioelectronics, energy conversion, and biomedicine^7–9^. In the field of tissue engineering, the incorporation of graphene and its derivatives into polymer has become an important way of manufacturing artificial compound scaffolds for tissue repair^10–12^. In this way, GBM improve the physical and mechanical characteristics as well as the general manifestation of the polymeric substrate by imparting significant cues such as electrical conductivity and mechanical reinforcement of synthetic scaffolds. This is very vital to electro-responsive tissue regeneration, such as nerves, cardiac, and skeletal muscles^13–16^. In addition to the unique properties, the biocompatibility of biomaterials should be considered carefully.

The biosafety profile of GBM has caught huge attention and remains a controversy in some studies. Different evaluation systems may yield different outcomes. Some claimed that graphene and its derivatives possessed huge potential in biological and medical fields. Previous literature reported that graphene could regulate cell attachment, migration, differentiation, and viability^17^. The GO displayed antibacterial ability by disrupting *E. coli* bacterial cell membranes, decreasing the viability by over 90%, and elucidating strong oxidative stress^18^. It further impaired membrane integrity by degrading phospholipids^19^. Polyvinyl-*N*-carbazole-GO (containing 3% GO) could even reduce the metabolic activity and induce cellular apoptosis by encapsulating bacterial cells^20^. In lung epithelial cells and fibroblasts, 50 μg mL^−1^ GO nanoparticles might induce minor toxicity and insignificant cell death^21^. Graphene, especially few-layer derivatives, exerted varied effects on neuronal cells. The expression level of caspase 3 was significantly increased in PC12 neuronal cells at the presence of 10 μg mL^−^^1^ few-layer graphene (FLG) that led to mitochondrial dysfunction and oxidant insults^22^. In contrast, another group found that FLG was quite biocompatible in three cell species, PC12 cells, oligodendroglial cells and osteoblasts^23^. These researches reported preliminary findings in cellular levels and resulted in huge deviations in GBM biocompatibility and other biological effects.

Even for in vivo evaluation, previous researches discussed the GBM biosafety via inhalation or intravenous injection, and found no prominent short-term toxicity^24^. Nevertheless, 1% graphene solution caused lung damage and inflammatory reactions with bioaccumulation and granuloma deposition in major functioning organs^25^. It seems more controversial in terms of in vivo application of GBM. Yang et al.^26^ found that polyethylene glycolylated nanographene sheets caused insignificant toxicity at the tested dose (20 mg kg^−1^) to the experimental mice during 3 months and were gradually cleared by both renal and fecal excretion. In contrast, Krajnak et al.^27^ reported that pathological changes and dysfunctions occurred after exposure to 40 μg graphene nanoparticles in the vascular/renal function in a load and form-dependent style in a mouse study. Therefore, it is vital and urgent to investigate the biological profiles of GBM comprehensively, especially in the nervous system. Our group previously reported the effects of polydopamine and RGD modified single-layered, multilayered graphene on improving Schwann cell (SC) proliferation and inducing myelination and axonal extension in a lengthy rat sciatic nerve defect model at 18 weeks after injury^28^. These findings preliminarily indicate that GBM promisingly facilitate neuronal repair after surface modification. However, the real biocompatibility of GBM alone is not clear in a long-term evaluation (over 1 year). Moreover, graphene can increase glial cell and SC viability in the central nervous system (CNS) and peripheral nervous system (PNS), respectively; Nevertheless, it has not been investigated whether GBM affect both nervous systems simultaneously because the CNS shows responsive neural plasticity after PNS injury. It seems exceptionally vital to dig into the deeper correlation in the neuronal regulation between CNS and PNS in peripheral nerve regeneration.

To solve these issues, the present study will lay emphasis on the long-term biological characteristics of graphene-based nanoscaffolds (GBN) in the PNS. In addition, interactions between PNS and CNS will also be investigated thoroughly to reflect the influence of GBN on neuronal activity and the microenvironment.

## Results

### Toxicity effect of GBN in a peripheral nerve defect

**Fig. 1:**
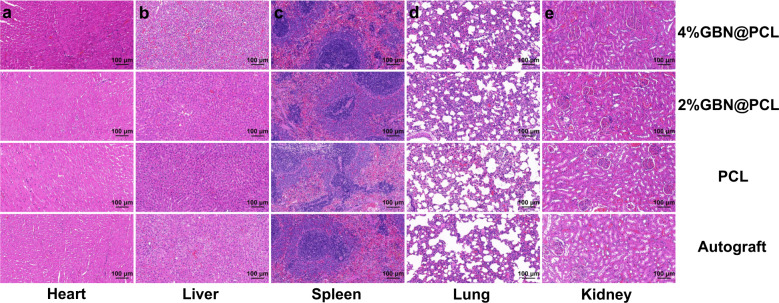
Toxicity effect of GBN in a peripheral nerve defect. Histology of major functioning organs (heart, liver, spleen, lung, and kidney) after 18-month scaffold or autologous nerve graft implantation in vivo using HE staining (**a**–**e**).

We investigated the toxicity effect of GBN in a lengthy sciatic nerve defect model over 18 months in vivo. The histological results of the major functioning organs reflected that no prominent morphological changes occurred in any of the heart, liver, spleen, lung, or kidney due to toxic insults from GBN (Fig. 1).

### Structural repair of peripheral nerves by GBN

**Fig. 2:**
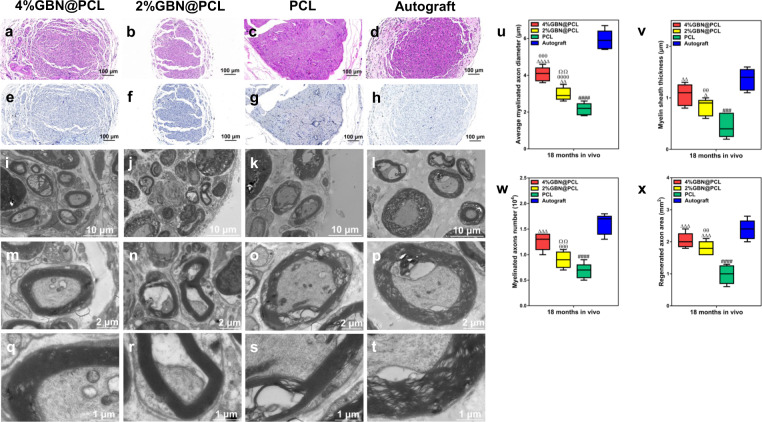
Structural repair of peripheral nerves by GBN. Morphology of sciatic nerve sections using HE (**a**–**d**), TB (**e**–**h**) staining, and transmission electron microscopy (**i**–**t**). Average myelinated axon diameter (μm) (**u**). Myelin sheath thickness (μm) (**v**). Myelinated axons number (10^4^) (**w**). Regenerated axon area (mm^2^) (**x**). ^Δ^ represents *p* < 0.05, ^ΔΔ^ represents *p* < 0.01, ^ΔΔΔ^ represents *p* < 0.001, ^ΔΔΔΔ^ represents *p* < 0.0001 in comparison with PCL; ^###^ represents *p* < 0.001, ^####^ represents *p* < 0.0001 for PCL vs. autograft. ^θθ^ represents *p* < 0.01, ^θθθ^ represents *p* < 0.001, ^θθθθ^ represents *p* < 0.0001 in comparison with autograft. ^ΩΩ^ represents *p* < 0.01 for 4%GBN@PCL vs. 2%GBN@PCL. The center line, bounds of box, and whiskers were displayed in the figures, respectively.

The peripheral nerve repair is measured by a few aspects, such as nerve structure, electrophysiology, and motor functions. There was no neuroma formation or persistent wound infection over 18 months after the injury. The myelin sheath and axons were carefully observed and evaluated on their diameters, thickness and total number, and areas. These parameters were higher in the autograft group than the graphene-loaded scaffold group, and the lowest in the polycaprolactone (PCL) group (Fig. 2a–h). Except for the average axon diameter, there was no significant difference between 4%GBN@PCL scaffold and autograft groups (Fig. 2i–t).

### Glial and neural expression of peripheral nerves and spinal cords by GBN

**Fig. 3:**
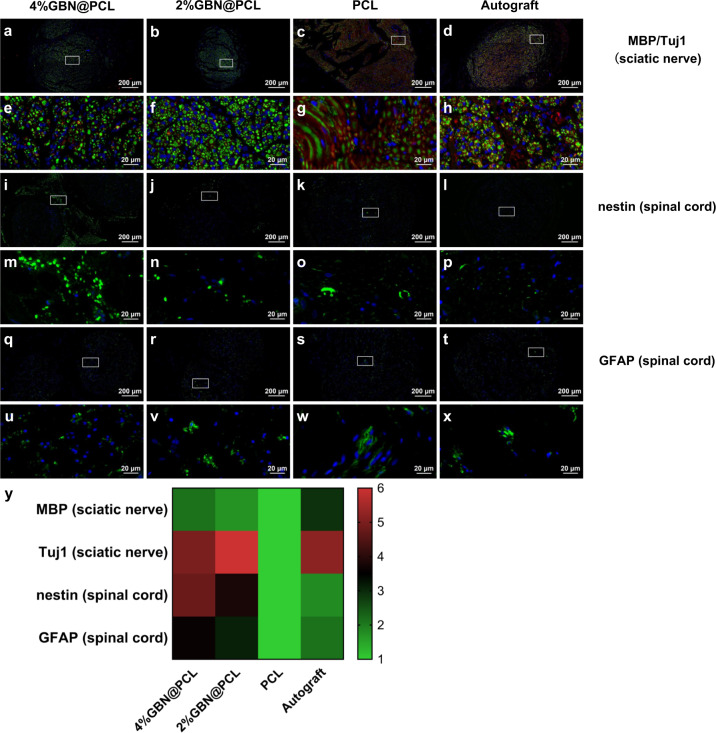
Glial and neural expression of peripheral nerves and spinal cords by GBN. MBP/Tuj1 triple immunostaining of the regenerated sciatic nerve sections (**a**–**h**). Red fluorescence, MBP; Green fluorescence, Tuj1; Blue fluorescence, nuclei. Nestin (**i**–**p**) and GFAP (**q**–**x**) immunostaining of the spinal cord. Green fluorescence, Nestin/GFAP; Blue fluorescence, nuclei. Relative expression levels of MBP and Tuj1 of the sciatic nerves, and nestin and GFAP of the spinal cord (**y**).

Myelin basic protein (MBP) expression levels were higher in the 4% and 2% layered graphene-loaded PCL scaffold groups than the PCL scaffold control group. Nevertheless, the MBP expression level was markedly higher in the autograft nerve (Fig. 3a–h). The immunofluorescent staining β-III-tubulin (Tuj1) results showed that its expression levels were similarly high among autograft and graphene-loaded PCL groups, and in contrast, much lower in the PCL control group (Fig. 3a–h). In addition, nestin and glial fibrillary acidic protein (GFAP) expression levels were relatively higher in the spinal cord tissue from graphene scaffold groups (Fig. 3i–y).

### Angiogenesis in the peripheral nerves by GBN

**Fig. 4:**
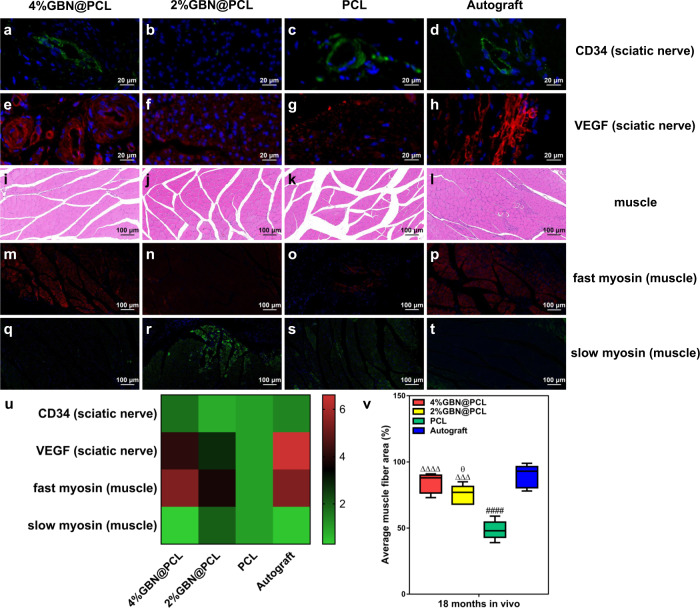
Angiogenesis in the peripheral nerves and muscle atrophy alleviation by GBN. CD34 (**a**–**d**) and VEGF (**e**–**h**) immunostaining of the regenerated sciatic nerve sections. Morphology of the gastrocnemius muscle using HE staining (**i**–**l**). Green fluorescence, CD34; Red fluorescence, VEGF; Blue fluorescence, nuclei. Fast myosin (**m**–**p**) and slow myosin (**q**–**t**) immunostaining of the gastrocnemius muscle. Red fluorescence, fast myosin; Green fluorescence, slow myosin; Blue fluorescence, nuclei. Relative expression levels of CD34 and VEGF of the sciatic nerves, and fast myosin and slow myosin of the muscles (**u**). Average muscle fiber area (%) (**v**). ^ΔΔΔ^ represents *p* < 0.001, ^ΔΔΔΔ^ represents *p* < 0.0001 in comparison with PCL; ^####^ represents *p* < 0.0001 for PCL vs. autograft. ^θ^ represents *p* < 0.05 in comparison with autograft. The center line, bounds of box, and whiskers were displayed in the figures, respectively.

We evaluated the endothelial cell activity by measuring CD34 and vascular endothelial growth factor (VEGF) protein expression levels. The angiogenesis status is significantly higher in the 4% and 2% graphene-based PCL scaffolds than PCL groups. The CD34 expression level was relatively higher in the 4%GBN@PCL group than autograft (Fig. 4a–h).

### Locomotor recovery of the peripheral nerves by GBN

**Fig. 5:**
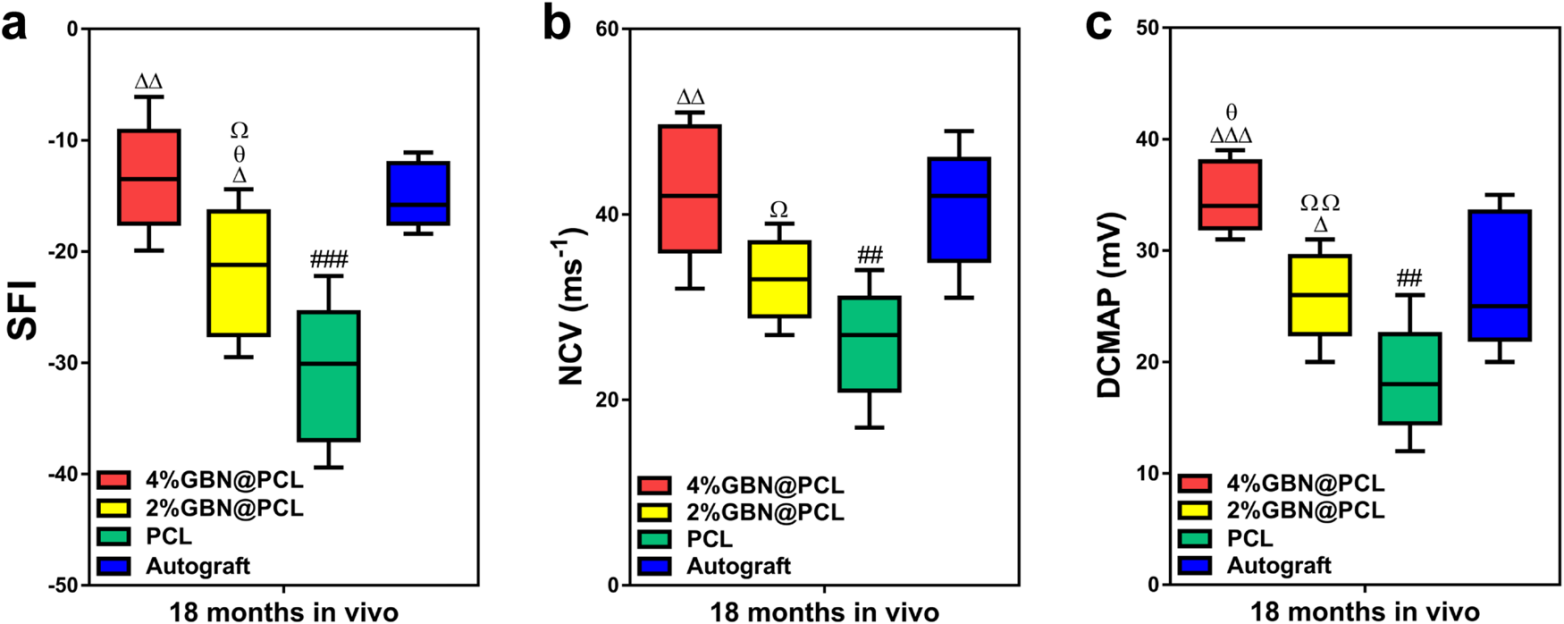
Locomotor and electrophysiology performances of the peripheral nerves by GBN. SFI (**a**). NCV (ms^−^^1^) (**b**). DCMAP (mV) (**c**). ^Δ^ represents *p* < 0.05, ^ΔΔ^ represents *p* < 0.01, ^ΔΔΔ^ represents *p* < 0.001 in comparison with PCL; ^##^ represents *p* < 0.01, ^###^ represents *p* < 0.001 for PCL vs. autograft. ^θ^ represents *p* < 0.05 in comparison with autograft. ^Ω^ represents *p* < 0.05, ^ΩΩ^ represents *p* < 0.01 for 4%GBN@PCL vs. 2%GBN@PCL. The center line, bounds of box, and whiskers were displayed in the figures, respectively.

In graphene-loaded PCL scaffolds, a higher value was seen in the muscle fiber area, though the muscle structure was the best in the autograft group (Fig. 4m–v). The results further showed that fast myosin expression levels were higher in the GBN groups (comparable to autograft) than in the PCL control group. The slow myosin expression level was just on the contrary (Fig. 4m–v). Furthermore, we evaluated the locomotor function by sciatic function index (SFI). It was the highest in the 4% graphene-loaded PCL group, and the lowest in the PCL group (Fig. 5a).

### Electrophysiological recovery of the peripheral nerves by GBN

The electromyography (EMG) was positioned on the injury side and then we evaluated nerve conducting velocity (NCV) and distal compound motor action potential (DCMAP) values, respectively. The 4% graphene-loaded PCL group showed a remarkably higher NCV level than other scaffold groups and comparable to autograft (Fig. 5b). DCMAP level was in consistent with NCV findings (Fig. 5c).

## Discussion

The strong C–C bond determines the excellent mechanical characteristics of graphene and its derivatives due to the perfect planar nature. The addition of graphene to polymeric materials is a great reinforcement to the compound scaffold^29^. PCL is one of the most common polymer materials used for neural substrates^30,31^. It is biocompatible and its biodegradation products are also toxic-free in the living body. Moreover, PCL provides certain mechanical stability with its high stiffness and relatively low degradation rate^32,33^. Consequently, the composite scaffold guarantees long-term in vivo support of structural integrity.

The biological application of GBM has resulted in huge attention on their biocompatibility and tissue toxicity both in vitro and in vivo. Like other carbon-based materials, graphene is not biodegradable under most conditions. However, it may be degraded at the presence of activated, degranulating neutrophils, by erythrophagocytosis in liver Kupffer cells, or by photodegradation in normal (HEK-293) and cancerous (HeLa and HepG2) human cells^34–46^. Graphene may cause different levels of tissue damage in organs (such as the lung)^37^. In the nervous system, previous researches showed widely divergent outcomes in various settings. Bianco et al.^38^ reported that graphene fabricated by chemical vapor deposition induced apoptotic effects, and an increased level of lactate dehydrogenase, and the formation of reactive oxygen species in neurons. Agarwal et al.^39^ found that single-layered graphene crosslinked collagen cryogel at 0.5% concentration was beneficial to the proliferation of bone marrow mesenchymal stem cells in vitro. However, the potential toxicity of GBN was rarely discussed in previous studies in vivo. Therefore, it is important to understand the real situation of the long-term performance of graphene nanomaterials in vivo for potential clinical application. In the meantime, the PCL degrades slowly in the physiological environment, from several months to years^40^. Consequently, we investigated the toxicity effect of GBN in a lengthy sciatic nerve defect model over 18 months in vivo. The findings indicate that relatively low concentration and loading of GBN (4% in the PCL scaffold in this study) may be biocompatible as it exerts no appreciable toxicity to the liver, kidney, heart, lung, or spleen in the long-term repair of peripheral nerves in vivo. The toxicity of nanomaterials, especially GBN concerns researchers due to the harm on major functioning organs and consequent hepatic and renal failure. It prevents the possible translational application of GBN materials in future clinical work. Our findings preliminarily confirm the relative biosafety profile of GBN in a long-term murine model.

Then, the biologically regenerative effects of GBN were thoroughly investigated for peripheral nerve injury (PNI) because these effects were also representative for evaluating bioactive materials in nerve tissue engineering. Axon and myelin were repaired well after graphene-based scaffold implantation. It implies a certain reparative capacity of GBN in a lengthy nerve defect. Although autologous nerve transplantation is the gold standard for treating injured peripheral nerves, its therapeutic effect is limited on the large defect so that additional alternative therapies are necessary^41^.

MBP is an important marker for myelin formation from SCs in the PNS^42^. The MBP expression level was positively associated with the development of the major dense line, and the compacted myelin sheath failed to form without the anchoring of the lipophilin particles to the myelin sheath^43^. In addition to myelin maturation evaluation by MBP, Tuj1 is a major neuronal protein and reflects axonal viability and regeneration after sciatic nerve injury^44^. The myelin sheath enwraps the axon tightly and these two important structures contribute to the healthy and functional peripheral nerve. The MBP and Tuj1 expression levels imply that GBN repair myelin and axons by recreating a pro-healing condition of the injured nerves.

In addition, the feedback of PNI on CNS was also investigated in detail. The spinal cord is directly influenced by PNI and starts to degenerate and form pathological plasticity immediately^45^. The spinal cord neurogenesis after PNI was measured and indicated by nestin and GFAP expression. Nestin is a class VI intermediate filament protein, which is expressed both in neuronal and glial progenitors and their common precursors^46^. The nestin-positive cells appear in the spinal cord following different injuries to the local site and distal peripheral nerves. GFAP is a class III intermediate filament protein and is widely expressed in astrocytes in the CNS^47^. Although previous researches have shown the reparative potential of GBN in CNS and PNS injuries, respectively, this study evaluates the multiple manipulations of central and peripheral neuronal activities by GBN in the severe PNI. The findings indicate a notable GBN-dependent increase in glial expression in the dorsal root ganglion, spontaneous ectopic activity in primary afferent fibers, neuronal sprouting, and rearrangement of axonal termination in the spinal cord after severe PNI. GBN can improve myelin extension and axon outgrowth by inducing a dual activation of central and peripheral neurons after PNI. Traditionally, the activation of astrocytes was considered as a pathological response to injury. However, many studies have shown that the mutual interaction between CNS and PNS is a potential sign of regeneration and is beneficial to neuronal proliferation, migration, and differentiation^48–50^. The simultaneous regulation of graphene-based scaffolds on PNS and CNS contributes to a better understanding on regenerative mechanisms of remyelination and axonal restoration.

An ideal microenvironment requires persistent support and transport of nutrients and energy. In PNI, the vessel regrowth and angiogenesis are of primary significance. A number of studies claimed a pro-angiogenic effect of graphene in different types of tissue damage^51–53^. However, their effects could not match the autograft nerves, at either in vitro settings or a relatively short-term in vivo period. In this study, we confirm that GBN may alleviate injury-related environment by restoring nutrient supply and stimulating vessel regrowth within regenerated areas.

The neuronal function is evaluated by both locomotor and electrophysiological activities^54^. The gastrocnemius muscle morphology and viability were extensively analyzed after PNI. In addition, muscle viability and proliferative status were evaluated using fast and slow myosin protein staining. These proteins represent proliferative and apoptotic muscle cells, respectively^55^. The findings show that muscle structure atrophy is alleviated and muscle viability is increased significantly. As to the electrical transduction evaluation, we evaluated and confirmed improved NCV and DCMAP values from the GBN scaffold, respectively. Based on the results, we think GBN significantly restore the locomotor and electrophysiological functions of the hind limb after severe PNI.

The appropriate evaluation of the biosafety of biomaterials is of huge significance, because the complex materials are positioned inside living tissues or organs and will stay for a lengthy term in the body. “Biocompatibility” refers to a biomaterial that is compatible with a living system by inducing insignificant toxicity or injuries. In addition, the biomedical scaffold is expected to induce a beneficial cue for tissue–biomaterial reaction in the body^56^. Graphene nanofamily has long been discussed and evaluated as to its potential biological applications, especially in the biomedical field. However, the real biocompatibility of GBN is uncertain^57^. There are many researches about the in vitro cytotoxicity of GBM in different disease settings. Nevertheless, this cannot mimic the real scenario of disease progression in the living system. Therefore, we performed the preclinical assessment of layered graphene-loaded PCL scaffold in the long-term regeneration of severe PNI in vivo. In addition to confirming potentially low toxicity and immune insult of the implants to the local site and main functioning organs (heart, liver, spleen, lung, and kidney), we further analyzed the regenerative capacity of GBN on rebalancing and restoring a pro-healing microenvironment for injured neurons and peripheral nerves.

GBN scaffold implants will be useful in many clinical situations. Their interaction with different vital supporting cells should be analyzed carefully in nerve regeneration. This will help to increase understanding of the mechanisms by GBN-dependent repair. In addition, researchers should put more efforts on evaluating potential biological and biocompatible profiles of GBN in large animal models in the future before clinical practice. In summary, the graphene-loaded PCL scaffold provides a biocompatible and biologically regenerative microenvironment for severe PNI after 18 months in a murine model. It exerts multiple pro-healing effects on myelination and axonal regeneration, possibly via dual regulation and activation of SCs and astroglia in the central and peripheral nervous systems. Furthermore, the graphene-loaded PCL scaffold significantly enhances the motor function of lower limbs and electrophysiological performances for nerve electrical transduction. This study implies that GBN may be advantageous for neuronal regeneration and will be useful for clinically translational application in the future.

## Methods

### Material fabrication

The layered graphene and PCL were purchased from Suzhou HENGQIU Technologies Co., Ltd. (China) and Perstorp (UK), respectively. The GBN was manufactured using a layer-by-layer casting technique. In brief, the layered graphene nanoparticles and PCL were dissolved in the dichloromethane and were injected onto a tubular mold from the inner to the outer layer. Then, microneedles were applied for adding aligned pores in the scaffold surface.

### Animal surgery

Sprague Dawley (SD) rats (male, 150–200 g, *n* = 5/group, Shanghai Xipuerbikai Laboratory Animal Company) were used for in vivo evaluation of the biological profiles of GBN. SD rats were randomly allocated into four groups: 4% layered graphene-loaded PCL scaffold, 2% layered graphene-loaded PCL scaffold, PCL scaffold, and autograft. They were kept in the specific pathogen-free animal house and were provided with enough water, food, leisure music, and free activity. The rats were anesthetized using sodium pentobarbital (provided by the animal experiment center) intra-peritoneally. The right thigh was exposed and the 18-mm sciatic nerve was dissected. The proximal and distal nerve stumps were reconnected by a scaffold or an autologous nerve segment. None of the animals suffered from infection, foot ulcer, death, or other complications after model establishment. Animal care and use were authorized by the Animal Ethics Committee of Shanghai Jiao Tong University (SJTU, No. A2017072).

### Walking track analysis and withdrawal latency

The locomotor function was evaluated at 18 months after surgery via walking track analysis by measuring the distance between the first toe and the fifth toe (TS), the third toe to the heal (PL), and the second toe to the fourth toe (IT). Experiment side (E) and normal side (N) were compared according to the formula, SFI = (−38.3 × (EPL − NPL) ÷ NPL) + (109.5 × (ETS − NTS) ÷ NTS) + (13.3 × (EIT − NIT) ÷ NIT) − 8.8. SFI values are positively correlated with functional performance. As to sensory performance, withdrawal latency was evaluated using a paw withdrawal apparatus (Hargreaves Model 390, USA).

### Electrophysiological experiment

Electrophysiological evaluation was carried out at 18 months after surgery under proper anesthesia with sodium pentobarbital (provided by the animal experiment center) of the rats. The bipolar electrodes were fixed at the distal and proximal nerve stumps in addition to another electrode at the belly of the gastrocnemius muscle for EMG under electrical stimulation. NCV was used to measure the conducting velocity between the two electrodes and DCMAP was used to reflect the excitability of the nerve fibers.

### Tissue harvest

All reagents were purchased from Sigma unless specified. All rats were sacrificed at 18 months post-injury. The major functioning organs, including heart, liver, spleen, lung and kidney, regenerated sciatic nerve, gastrocnemius muscle, and dorsal root of spinal cord tissues were harvested carefully from each group. After euthanasia using an overdose of sodium pentobarbital (provided by the animal experiment center) for the rats, we shaved the surgical area and incised the injured site to expose the nerve and the scaffold. Then, we removed the scaffold and the regenerated nerve inside the scaffold by cutting off two ends, harvested the dorsal root of the spinal cord, and then rinsed the samples in the phosphate buffer saline (PBS) three times. Next, we dissected the gastrocnemius muscle from the injured leg with a mosquito clamp and scalpel without tearing muscle fibers, and rinsed the muscle sample in the PBS three times. Afterward, we incised the abdominal and thoracic cavity to separate and fetch the liver, spleen, kidney, lung, and heart carefully, and rinsed the samples in the PBS three times.

### Hematoxylin–eosin (HE) staining

All reagents were purchased from Sigma unless specified. The sciatic nerves, gastrocnemius muscle, and organs (heart, liver, spleen, lung, and kidney) were fixed in 4% paraformaldehyde fix solution overnight, and then were rinsed, and dehydrated in 75%, 80%, 95%, 95%, 100% and 100% ethanol solution for 1 h, respectively. Then, the samples were waxed at around 56 °C, embedded in paraffin, and sliced in order. The paraffin sections were dewaxed using dimethylbenzene and ethanol, respectively, and then they were immersed in hematoxylin for 8 min, and in 0.6% ammonia before running-water rinsing. Then, sections were stained in the eosin for 3 min and were dehydrated using ethanol before mounting.

### Toluidine blue (TB) staining

All reagents were purchased from Sigma unless specified. The sciatic nerves were fixed in 4% paraformaldehyde fix solution overnight, and were rinsed in PBS. Then, sections were dewaxed, and immersed in TB solution for 30 min at 50 °C, and were dehydrated in 75%, 90%, and 100% ethanol solution, respectively. The samples were processed using dimethylbenzene before mounting.

### Immunofluorescence

All reagents were purchased from Sigma unless specified. The sciatic nerves, spinal cord, and gastrocnemius muscle were fixed in 4% paraformaldehyde fix solution overnight, and then were rinsed, and dehydrated in 75%, 80%, 95%, 95%, 100%, and 100% ethanol solution for 1 h, respectively. Then, the samples were waxed at around 56 °C, embedded in paraffin, and sliced in order. Then, the slices were blocked by 5% bovine serum albumin (Gibco, Shanghai, China) and incubated with primary antibodies overnight and secondary antibodies for 2 h at room temperature, respectively. Primary antibodies included anti-GFAP (1:400, Abcam, ab7260, Shanghai, China), anti-nestin (1:500, Abcam, ab254048, Shanghai, China), anti-CD34 (1:500, Abcam, ab185732, Shanghai, China), anti-VEGF (1:500, Abcam, ab81289, Shanghai, China), anti-Tuj1 (1:400, Abcam, ab18207, Shanghai, China), anti-MBP (1:400, Abcam, ab40390, Shanghai, China), anti-fast type myosin (1:300, Abcam, ab228727, Shanghai, China), and anti-slow type myosin (1:300, Abcam, ab11083, Shanghai, China).

### Transmission electron microscopy (TEM)

All reagents were purchased from Sigma unless specified. The ultrathin sciatic nerve samples were immersed in 2.5% glutaraldehyde solution for 2 h at 4 °C, rinsed in PBS for three times, and fixed in 1% osmic acid for 2 h. Then, the samples were rinsed in PBS again and underwent dehydration by acetone (50%, 70%, 90%, and 100%) for 1 h. After that, the samples were embedded in Epon812 at 37 °C overnight. Finally, after double staining with 3% uranium citrate, the ultra-structure of sciatic nerve sections was evaluated.

### Microscopy and evaluation

Sections for HE and TB assays were observed under an optical microscope (Zeiss, Germany) and random vision fields were pictured from evaluation. Sections for immunofluorescence assays were observed under an immunofluorescence microscope (Leica, USA) and random vision fields were pictured from evaluation. The ultra-structure of sciatic nerve sections was observed under a transmission electron microscope (HITACHI, Japan) at 80-kV voltage. Different magnification was chosen for observation, ×1000, ×3000, and ×8000 by selecting random vision fields. The number and area of myelinated axons were observed by HE and TB staining and calculated by identifying positive areas using image pro plus 6.0 software. The diameter of the myelinated fiber and myelin sheath thickness were observed by TEM calculated by identifying axon fiber and myelin sheath structures using image pro plus 6.0 software. The average muscle fiber area was observed by HE staining and calculated using image pro plus 6.0 software. Relevant expression levels of different proteins were calculated based on the immunofluorescent intensity measured using image pro plus 6.0 software.

### Statistical analysis

The statistical analysis was carried out by GraphPad 7.0 Software (San Diego, CA); the results were displayed as the mean ± standard deviation. A *p* < 0.05 by one-way analysis of variance (ANOVA) was statistically significant in this study.

### Reporting summary

Further information on research design is available in the Nature Research Reporting Summary linked to this article.

## Supplementary information

Reporting Summary

## Data Availability

The data that support the findings of this study are available in this article or from the corresponding author upon reasonable request.
